# Intracerebral transplantation of HLA‐homozygous human iPSC‐derived neural precursors ameliorates the behavioural and pathological deficits in a rodent model of ischaemic stroke

**DOI:** 10.1111/cpr.12884

**Published:** 2020-07-26

**Authors:** Jeong‐Eun Noh, Seung‐Hun Oh, Suji Lee, Soohyeon Lee, Young Hoon Kim, Hyun Jung Park, Ji Hyeon Ju, Hyun Sook Kim, Ji Young Huh, Jihwan Song

**Affiliations:** ^1^ Department of Biomedical Science CHA Stem Cell Institute CHA University Seongnam‐si Korea; ^2^ Department of Neurology CHA Bundang Medical Center CHA University Seongnam‐si Korea; ^3^ Department of Internal Medicine Seoul St. Mary’s Hospital Institute of Medical Science The Catholic University of Korea Seoul Korea; ^4^ Department of Laboratory Medicine CHA Bundang Medical Center CHA University Seongnam‐si Korea; ^5^ iPS Bio, Inc. Seongnam‐si Korea

## Abstract

**Objectives:**

Human‐induced pluripotent stem cells (hiPSCs) are a promising cell source for treating ischaemic stroke. Although autologous hiPSCs provide the advantage of avoiding immune rejection, their practical limitations, such as substantial amount of time and costs to generate individual iPSC lines, have hampered their widespread application in clinical settings. In this study, we investigated the therapeutic potential of neural precursor cells derived from human HLA‐homozygous induced pluripotent stem cells (hiPSC‐NPCs) following intracerebral transplantation into a rodent model of middle cerebral artery occlusion (MCAo).

**Materials and Methods:**

We differentiated a GMP‐grade HLA‐homozygous hiPSC line (CMC‐hiPSC‐004) into neural precursor cells for transplantation into rats at the subacute stage of ischaemic stroke (ie at 7 days after the induction of MCAo). To investigate functional recovery, the transplanted animals were subjected to five behavioural tests, namely the rotarod, stepping, mNSS, staircase and apomorphine‐induced rotation tests, for up to 12 weeks, followed by histological analyses.

**Results:**

We observed that the hiPSC‐NPC transplantation produced significant behavioural improvements. At 12 weeks post‐transplantation, a high proportion of transplanted cells survived and had differentiated into MAP2^+^ mature neurons, GABAergic neurons and DARPP32^+^ medium spiny neurons. The transplanted cells formed neuronal connections with striatal neurons in the host brain. In addition, hiPSC‐NPC transplantation gave rise to enhanced endogenous repair processes, including decreases of post‐stroke neuroinflammation and glial scar formation and an increase of proliferating endogenous neural stem cells in the subventricular zone as well as the perilesional capillary networks.

**Conclusions:**

These results strongly suggest that HLA‐homozygous hiPSC‐NPCs may be useful for treating ischaemic stroke patients.

## Graphical abstract

We transplanted neural precursor cells derived from HLA homozygous hiPSC into the subacute‐stage ischemic stroke rats and observed significant behavioral improvements in the rotarod, stepping, mNSS, staircase and apomorphine‐induced rotation tests. At 12 weeks post‐transplantation, a high proportion of transplanted cells survived and were differentiated into MAP2^+^ mature neurons, GABAergic neurons and DARPP32^+^ medium spiny neurons. Transplanted cells formed a neuronal connection with striatal neurons in the host brain. They also gave rise to enhanced endogenous repair processes, including the decrease of post‐stroke neuroinflammation and glial scar formation and the increase of proliferating endogenous neural stem cells in the subventricular zone as well as the perilesional capillary networks. These results strongly suggest that HLA‐homozygous hiPSC‐NPCs may serve as a useful candidate to treat ischemic stroke patients in the future.

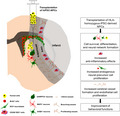

## 1 INTRODUCTION

Ischaemic stroke is the most common form of stroke, accounting for approximately 85% of stroke cases. It is caused by the blockage of blood flow in the brain, resulting in the shortage of oxygen or nutrients, which causes brain cells to die. With the exception of thrombolytic therapy within 4.5 hours after stroke, there is no effective therapy for stroke beyond this therapeutic time window,^1^ and harnessing the potential of stem cells or other types of cell therapy to regenerate brain tissue lost due to stroke was regarded as being a long way off.^2^ However, in recent years, substantial efforts have been made to develop cell therapies for ischaemic stroke using stem cells from various sources.^3^, ^4^ The transplantation of stem cells can improve behavioural impairments in animal models of stroke^3^, ^5^, ^6^, ^7^ through several mechanisms, including immune modulation,^8^, ^9^ neuroprotection,^10^, ^11^, ^12^, ^13^ stimulation of neurogenesis^14^, ^15^, ^16^ and angiogenesis,^8^, ^13^, ^14^ as well as neural replacement.^7^, ^17^, ^18^, ^19^ Among various stem cell sources, neural precursor cells (NPCs) are among the most attractive for stem cell therapy because they can differentiate into various different neural lineages that are needed for the replacement of cells in the stroke‐damaged brain. NPCs are obtained from aborted foetal brain tissues or are derived from human embryonic stem cells (hESCs). However, ethical concerns and allogeneic rejection are the critical barriers for the clinical application of these cell sources. The discovery of human‐induced pluripotent stem cells (hiPSCs) has provided a therapeutic opportunity to use the patient's own somatic cells in many diseases. Although hiPSCs are a powerful source for cell therapy without the risk of immune rejection, in reality, it would be extremely expensive and labour‐intensive to generate autologous hiPSCs for personalized medicine. Moreover, in the case of autologous transplantation, individual iPSCs should meet the regulatory requirements each time when their clinical application is needed. In addition, autologous hiPSCs from diseased patients may carry the same genetic defect, which would reduce the therapeutic efficacy when they are used for cell therapy. Therefore, generating autologous iPSCs from each individual is not practical.

An alternative strategy is to make use of a human leucocyte antigen (HLA) haplotype donor to provide HLA‐matched materials to significant numbers of patients. In the clinical field of solid organ transplantation or hematopoietic stem cell transplantation, immunosuppression and HLA‐matching have been used to limit alloimmune responses.^20^, ^21^ HLA‐homozygous hiPSCs can reduce the need for immunosuppressive agents when transplanted into HLA‐matched patients. Therefore, the generation of HLA‐homozygous hiPSCs has opened up a new opportunity in the development of cell therapy because it can be utilized to treat a large number of patients with a relatively small number of well‐selected donors.^22^ Therefore, much progress has been made in the establishment of HLA‐homozygous hiPSC banks that can cover a significant proportion of the population in various countries including the United States,^23^ Japan^24^ and South Korea.^25^, ^26^

In this study, we investigated whether intracerebral transplantation of HLA‐homozygous hiPSC‐NPCs can improve behavioural and pathological deficits in a rodent model of stroke. If HLA‐homozygous hiPSC‐NPCs demonstrate therapeutic potential for functional recovery in an animal stroke model, it will provide a basis for the clinical application of HLA‐homozygous hiPSCs to treat stroke patients in the near future.

## 2 MATERIALS AND METHODS

### 2.1 Preparation of hiPSC‐NPCs

The hiPSC line, CMC‐hiPSC‐004, used in this study was as previously described.^25^ In brief, CMC‐hiPSC‐004 was generated from peripheral blood mononuclear cells (PBMCs) of an 18‐year‐old man. This hiPSC line contains the homozygous HLA types of A*33:03~B*44:03~DRB1*07:01, which corresponds to the third most common haplotype in South Koreans (ie 2.68% estimated frequency in the South Korean population).^26^ The hiPSC line was maintained in Stemfit^®^ Basic02 medium (Ajinomoto), supplemented with 100 ng/mL basic fibroblast growth factor (bFGF) and 10 µmol/L Y27632 (ROCK inhibitor), for about 7 days before being treated with TrypLE solution (Thermo Fisher Scientific) for 5 minutes at 37°C in a CO_2_ incubator. The dissociated cells were cultured in SFEBq medium, consisting of DMEM/F12 (Thermo Fisher Scientific) supplemented with 1% antimycotic‐antibiotics, 1% non‐essential amino acids (NEAAs), 0.1% beta‐MeOH, 20% Knockout™ SR, 10 µmol/L SB431542, 100 nmol/L LDN193189 and 3× ROCK inhibitor at 37°C in a CO_2_ incubator for neural induction. The cells were maintained in the SFEBq medium for 8 days. Embryoid bodies were dissociated in the neural precursor cell (NPC) medium, consisting of DMEM/F12 supplemented with 1:100 antimycotic‐antibiotics, 1:100 NEAA, sodium pyruvate, d‐glucose, l‐glutamine, 1:1000 beta‐MeOH, 1:50 B‐27 (without vitamin A), and 20 ng/mL bFGF in a dish coated with poly‐l‐ornithine and laminin (Figure S1A). We used accutase to split the cells for transplantation.

### 2.2 Neuronal differentiation and immunocytochemical analysis of hiPSC‐NPCs

To confirm the differentiation ability of NPCs derived from the CMC‐hiPSC‐004 line, we spontaneously differentiated them into mature neurons. The hiPSC‐NPCs derived from the above processes were passaged and the medium was changed to mature neuron medium, consisting of neurobasal A Medium, 1× GlutaMAX and 1× B27 supplement (Figure S1A). For differentiation, medium was supplemented with 20 ng/mL BDNF for mature neurons, 10 ng/mL BDNF and 0.5 µmol/L purmorphamine for GABAergic neurons, and 100 ng/mL SHH, 100 ng/mL FGF8 and 1 µg/mL cAMP for dopaminergic neurons. Next, morphological analysis and immunocytochemical staining using antibodies against NPCs and mature neurons were performed during the course of neural differentiation. The cells were fixed with 4% paraformaldehyde for 15 minutes and non‐specific binding was blocked with 0.1% Triton X‐100/PBS three times, followed by washing with 5% normal horse serum/PBS for 30 minutes. The specimens were incubated with primary antibodies at 4°C for 12 hours and then washed with PBS three times. Afterwards, secondary antibodies were applied. Nuclei were counter‐stained with 4,6‐diamidino‐2‐phenylindole (DAPI, 1:1000; Roche). The primary antibodies used in this study are described in Table S1. The secondary antibodies used included the goat anti‐mouse IgG Alexa 555 (1:250; Thermo Fisher Scientific), goat anti‐rabbit IgG Alexa 488 (1:250; Thermo Fisher Scientific) and goat anti‐mouse IgM Alexa 555 (1:250; Thermo Fisher Scientific).

### 2.3 Middle cerebral artery occlusion animal model

All animal experiments were performed in accordance with the CHA University IACUC (Institutional Animal Care and Use Committee) guidelines (IACUC150066). A stroke model was induced by transient middle cerebral artery occlusion (MCAo) for 90 minutes.^27^ Adult male Sprague Dawley rats (Orients) weighing 270‐300 g were used in this experiment. After anaesthesia with 1% ketamine (57.6 mg/kg) and xylazine (7.7 mg/kg) by intraperitoneal (i.p.) injection, the rats were maintained at a body temperature of 37 ± 1°C by placing them in a supine position on a heating pad. The right common carotid artery (CCA), external carotid artery (ECA) and internal carotid artery (ICA) were exposed, and a blunt‐ended silicon‐coated monofilament (4‐0; Ethicon, Pinewood) was inserted to occlude the middle cerebral artery (MCA) for 90 minutes before it was removed. The day after MCAo surgery, we performed acute neurological assessments (ie forelimb and hindlimb placement tests and circling behaviour test) to select the suitable stroke rat models. We selected animals with moderate neurological deficits for experiment (ie 2 or 3 points), in which 1 is severe and 5 is normal in the acute neurological assessment. In addition, before transplantation (ie 7 days after MCAo induction), we finally selected the rats with moderate to severe sensorimotor deficits [ie a score of 15 points or higher on the modified neurological severity scale (mNSS)] for the experiments. Among 52 rats, 12 were not used due to death before transplantation (n = 7) or mild neurological deficits (n = 5). Therefore, a total of 40 rats were used in this study.

### 2.4 Cell transplantation

To investigate the therapeutic effects of hiPSC‐NPCs, we designed the transplantation experiment by allocating the rats into three groups as follows: (a) Group 1 (Medium group): 2 µL of medium (n = 10), (b) Group 2 (Fibroblast group): 2 × 10^5^/2 µL of fibroblasts (n = 10), and (3) Group 3 (iPSC‐NPC group): 2 × 10^5^/2 µL of hiPSC‐NPCs (n = 10). At 7 days after MCAo induction, we transplanted the cells into two sites (1 × 10^5^ cells/µL each) as follows: (a) anterior–posterior (AP): +1.0 mm; medial‐lateral (ML): −2.5 mm; dorsal‐ventral (DV): −2 mm from the bregma, and (b) AP: +1.0 mm; ML: −2.5 mm; DV: −7 mm from the bregma. All animals were immunosuppressed with cyclosporine A (15 mg/kg; CKD Pharmaceuticals) intraperitoneally, starting from 1 day before transplantation and continuing every day throughout the study.

### 2.5 Retrograde tracer injection

At 12 weeks after transplantation, we stereotaxically injected 0.5 µL of 4% Fluoro‐Gold (FG) (Fluorochrome) into the globus pallidus (AP: −1.3 mm, ML: −3.4 mm, DV: −6.5 mm) on the ipsilateral side to MCAo.^28^ One week later, we sacrificed the animals and extracted their brains for histological analyses (n = 3 from each group).

### 2.6 5′‐Bromo‐2′‐deoxyuridine (BrdU) injection

To detect the endogenously proliferating stem cells,^29^ we injected 5′‐bromo‐2′‐deoxyuridine (BrdU) (50 mg/kg; Sigma‐Aldrich) intraperitoneally for 1 week at 12 hours intervals prior to sacrifice (n = 3 for each group).

### 2.7 Behavioural tests

We performed five tests to monitor the behavioural changes upon the transplantation of the hiPSC‐NPCs (n = 10 for each group). To reduce variation among the animals, the rats were trained for rotarod and staircase tests three times a day under the same conditions for 3 consecutive days before the induction of MCAo. For the baseline standards, rotarod, stepping and staircase tests were performed before MCAo (pre data), and all five tests were performed 1 day after MCAo (0W data), 1 week after MCAo (1W data) and weekly for 12 weeks.

#### 2.7.1 Rotarod test

We performed the rotarod test to investigate motor function and balance control.^30^ We measured the duration until the animal fell off the rotarod that was accelerated from 0 to 40 r.p.m. within a total of 120 seconds. This test was performed three times a day every week, and the mean time was calculated.

#### 2.7.2 Stepping test

We performed the stepping test to investigate sensory and motor functions.^31^ The experimenter held all animals in the same position and fixed one forelimb and two hindlimbs of each animal. The unfixed forelimb of the rat was allowed to touch the board (900 mm in length for 5 seconds) and move sideways slowly by the experimenter, first in the forward and then backward direction. Two forelimbs were measured alternately using the same method. The number of steps with which the rats placed their two forelimbs on the board was counted, and we then calculated the mean of the ratio of stroke‐affected forelimb vs. unaffected forelimb. The test was performed three times a day every week.

#### 2.7.3 Modified neurological severity score (mNSS) test

We performed the mNSS test to evaluate the neurological deficits of ischaemic stroke‐damaged rats after transplantation. This test is a composite of motor, sensory, beam balance and reflex tests^32^, ^33^, ^34^, ^35^ and is graded on a scale of 0‐28 (normal score: 0, maximal deficit score: 28). The test was performed every week.

#### 2.7.4 Staircase test

We performed the staircase test to evaluate the independent use of the forelimbs in 'site‐specific' skilled reaching and grasping tasks.^36^ Animals were pretrained prior to the experiment, to learn how to eat pellets placed on the concave holes using their forelimbs. Each rat was placed in the staircase apparatus with five pellets on the affected, left side for 15 minutes, and the number of pellets eaten by the rats was counted. The test was conducted once a day for three consecutive days biweekly.

#### 2.7.5 Apomorphine‐induced rotation test

We also performed the apomorphine‐induced rotation test, which can provide sensitive and rapid behavioural correlates of the substantia nigra.^37^ When the substantia nigra region was damaged, animals injected with apomorphine (1.0 mg/kg in saline containing 0.02% ascorbate; Sigma) were rotated towards the unaffected side. All animals were equipped with a harness with a thin steel wire that transferred the movement of the animal to electromechanical sensors. All animals were injected with apomorphine intraperitoneally and, starting 5 minutes later, the number of rotations within 60 minutes was counted. This test was performed at 0, 2, 4, 8 and 12 weeks.

### 2.8 Tissue preparation

At 12 weeks after the transplantation of hiPSC‐NPCs, all animals were anesthetized by the intraperitoneal injection of 1% ketamine (30 mg/kg) and xylazine hydrochloride (4 mg/kg), and then perfused transcardially with saline and 4% paraformaldehyde.^38^ Brains were extracted and post‐fixed overnight in 4% paraformaldehyde at 4°C, followed by transfer to 30% sucrose solution for 2 days until they sank. Brains were stored at −80°C after freezing in OCT compound (Lot No. 3801480; Leica). Brains were sectioned at 40 µm thickness coronally using a cryostat (Leica CM3050 S; Leica Microsystems) and stored in 24‐well plates until use.

### 2.9 Immunohistochemistry

Free‐floating brain sections were washed three times for 15 minutes in PBS, three times for 10 minutes in tPBS solution containing 0.3% Triton X‐100 (Sigma) and then blocked for 60 minutes in tPBS solution containing 5% normal horse serum (Vector Laboratories) at room temperature. Sections were incubated with primary antibodies, shown in Table S2, at 4°C for 12 hours. Subsequently, sections were washed five times for 10 minutes each in PBS and then incubated in the corresponding fluorescence‐conjugated secondary antibodies against each primary antibody for 90 minutes. The secondary antibodies used in this study were as follows: goat anti‐mouse IgG‐conjugated Alexa‐488 (1:250; Invitrogen), goat anti‐rabbit IgG‐conjugated Alexa‐488 (1:250; Invitrogen), goat anti‐mouse IgG‐conjugated Alexa‐555 (1:250; Invitrogen), goat anti‐rabbit IgG‐conjugated Alexa‐555 (1:250; Invitrogen) and donkey anti‐goat IgG‐conjugated Alexa‐555 (1:250; Invitrogen). Sections were then washed for 10 minutes in PBS and incubated in DAPI (1:500; Roche) stain for 30 minutes to label the cell nuclei. Fluorescence‐labelled sections were imaged using a confocal laser‐scanning microscope (Leica TCS SP5 II; Leica Microsystems). BrdU‐positive cells were detected by immunohistochemistry using an antibody against BrdU following denaturation of DNA in 1 mol/L HCl for 30 minutes at 45°C. The procedures for secondary antibody incubation, counter‐staining and confocal analysis were the same as described above.

### 2.10 Cell counting

All quantifications and analyses were performed as described previously.^19^, ^28^, ^39^, ^40^, ^41^ Typically, three coronal sections cut at 40 µm thickness (AP: +1.0, 0 and −1.0 mm) from each animal following double immunohistochemical staining were used. Stereological quantification of co‐labelled cells was performed in the region of interest (ROI) in the cortex and striatum of the ischaemic penumbra and boundary regions under a 40× objective from a confocal laser‐scanning microscope. To examine the survival and differentiation of transplanted human cells, we analysed 13 brain sections, starting from AP +1.5 mm to AP −1.5 mm in each animal, using a 40× objective of the confocal laser‐scanning microscope. Data are presented as the percentage of positive cells. To investigate the changes of inflammatory responses in the host brain following transplantation, we analysed five ROIs from the ischaemic boundary region using a 40× objective. Data are presented as the percentage of positive cells out of the total DAPI‐positive cells. To measure the changes of glial scar formation, we quantified five ROIs, adjacent to the ischaemic area of GFAP‐positive areas. The glial scar‐forming areas as well as their thickness were measured as previously described.^10^ Data are presented as the mean area (µm^2^)/ROI and the mean thickness (µm)/ROI.

To examine the changes of endogenous neurogenesis, we counted the proliferating cells in the three areas of the subventricular zone (SVZ). To do this, we counted the numbers for BrdU^+^ cells alone, DCX^+^ cells alone and BrdU^+^‐DCX^+^ co‐labelled cells at five ROIs within the ipsilateral SVZ wall and the data are presented as the percentage of positive cells out of DAPI‐positive cells. For the quantitative measurement of cerebral vessels, we counted RECA1^+^ vessels formed by endothelial cells at four ROIs in the ischaemic penumbra under a 10× objective lens of a light microscope (Nikon Eclipse E600) (n = 5 from each group). All cell counting analyses were performed using ImageJ software (NIH).

### 2.11 Infarct size measurement

Cresyl violet staining on 16 2‐µm‐thick coronal sections was performed to measure the final infarct size (n = 7 from each group). A total of eight serial sections were analysed in each animal. The infarct size was defined as a percentage of the intact contralateral hemisphere using the following equation: estimated infarct size (%) = [1 − (area of remaining ipsilateral hemisphere/area of intact contralateral hemisphere)] × 100. The areas of interest were measured using ImageJ software, and the values were summed for eight serial coronal sections per brain.

### 2.12 Statistical analysis

Statistical analysis of all experiments was performed using Prism software (version 8.0, GraphPad). Tissue analysis was performed using one‐way analysis of variance (ANOVA), and the behavioural performance was analysed using two‐way ANOVA. For multiple group comparisons, post hoc Tukey's *b* test was used. All data are presented as mean ± standard error of the mean. *P* values <.05 were considered statistically significant.

## 3 RESULTS

### 3.1 Neuronal differentiation of hiPSCs

Figure S1A outlines the experimental scheme for neuronal differentiation (Figure S1A). First, we differentiated hiPSCs into neural precursor cells (NPCs), which expressed the markers for NPCs such as Sox2, Nestin and Musashi (Figure S1B). Next, we observed their potential to differentiate into neurons and express various neuronal markers, such as Tuj1, GABA, TH and DARPP‐32 (Figure S1C). Tuj1^+^, GABA^+^ and TH^+^ cells were observed at 3 weeks after neural induction, whereas DARPP‐32^+^ cells were observed at 13 weeks after it. Additionally, we confirmed that the neurons derived from hiPSC‐NPCs showed immunoreactivity for SVP38 and PSD95 at 13 weeks after neural induction, indicating that the differentiated cells developed synaptic formation as they differentiated into mature neurons (Figure S1C).

### 3.2 Behavioural recovery following transplantation of hiPSC‐NPCs

**Figure 1:**
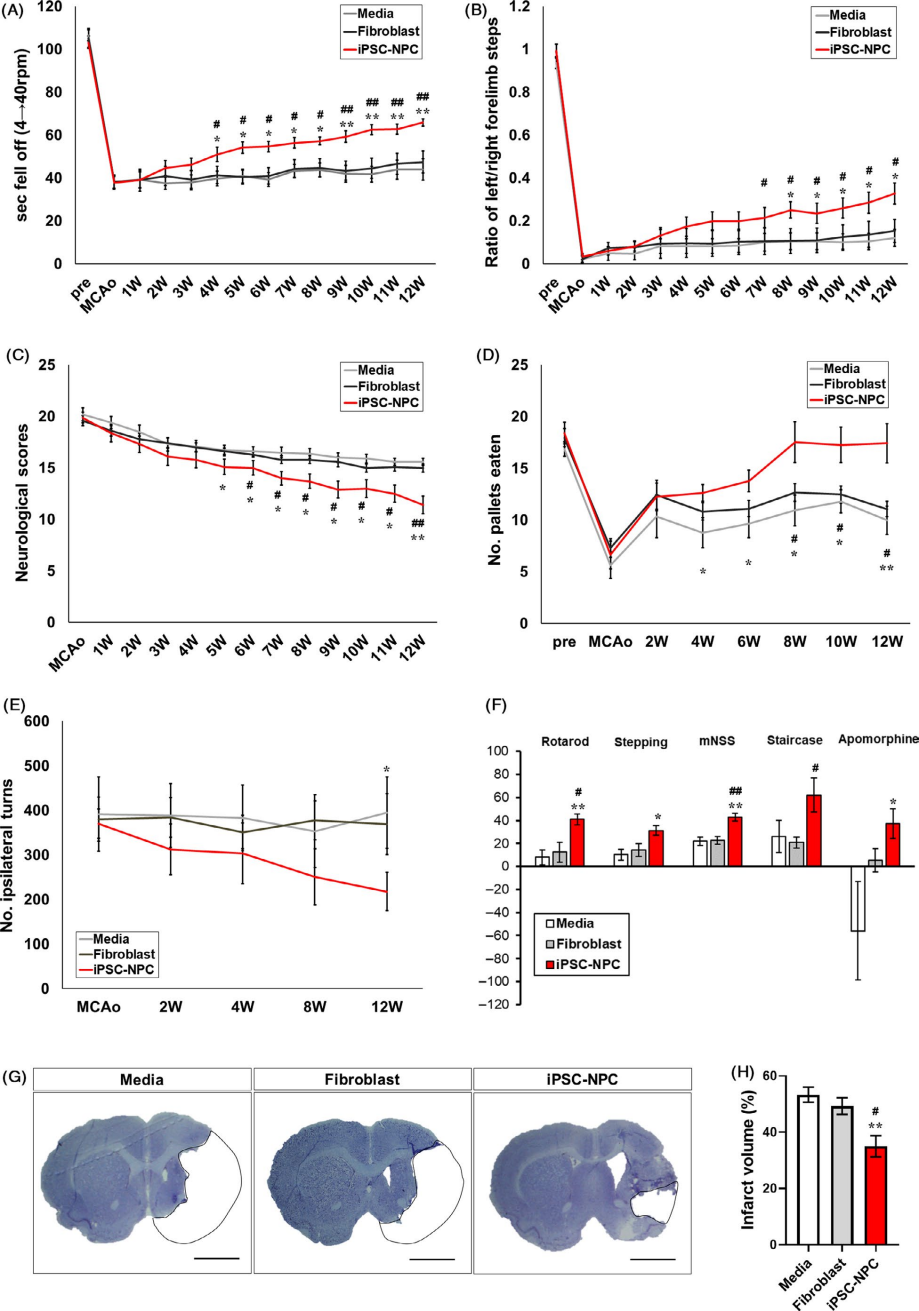
Behavioural tests following transplantation of hiPSC‐NPCs into MCAo rats. Rotarod test (A), stepping test (B), modified neurological severity score (mNSS) test (C), staircase test (D) and apomorphine‐induced rotation test (E) were performed during 12 wk after MCAo induction in the Medium, Fibroblast and iPSC‐NPC groups (n = 10 per group). F, The recovery rates of each behavioural test during 12 wk following transplantation. The recovery rate was defined as the percentage of the final score relative to the baseline score in each test. G, Representative image of cresyl violet staining. The area indicated by the dashed line indicates the infarct area. Scale bar = 100 µm. H, Quantitative analysis of infarct size in the Medium, Fibroblast and iPSC‐NPC groups (n = 7 per group). Data are expressed as the mean ± SEM. Statistical significance determined by two‐way ANOVA is shown as follows: for Medium vs iPSC‐NPC: *P < .05; **P < .01; for Fibroblast vs iPSC‐NPC: ^#^ *P *< .05; ^##^ *P *< .01

To investigate whether the transplantation of hiPSC‐NPCs can improve behavioural deficits caused by MCAo, we performed five tests for 12 weeks following the transplantation of hiPSC‐NPCs. The iPSC‐NPC group showed significant improvements in all five behavioural tests compared with the Medium and Fibroblast groups. On the rotarod test, the iPSC‐NPC group showed an increase in the time to fall from the rod, starting from 4 weeks, compared with the Medium and Fibroblast groups. This significant difference was maintained up to 12 weeks (Figure 1A). In the stepping test, the iPSC‐NPC group exhibited a significant behavioural improvement compared with the Medium and Fibroblast groups, from 7 weeks up to 12 weeks (Figure 1B). In the mNSS test, the iPSC‐NPC group showed significant reductions in the scores of neurological deficits from 5 weeks up to 12 weeks (Figure 1C). In addition, in the staircase test, the iPSC‐NPC group showed significant behavioural improvement from 4 weeks up to 12 weeks (Figure 1D). Moreover, in the apomorphine‐induced rotation test, the iPSC‐NPC group exhibited a significant improvement compared with the two control groups at 12 weeks (Figure 1D,E). Collectively, the findings revealed that the iPSC‐NPC group showed significant behavioural improvements from baseline in all five tests compared with the Medium group and the Fibroblast group (Figure 1F). In addition to functional recovery, the final infarct size of the iPSC‐NPC group (35.01 ± 3.45%) was significantly decreased compared with those of the Medium group (53.35 ± 2.47%) and the Fibroblast group (49.30 ± 2.73%) (Figure 1G,H). These results indicate that intracerebral transplantation of hiPSC‐NPCs in the subacute stage of ischaemic stroke restored functional deficits in rat stroke models.

### 3.3 Survival and engraftment of transplanted hiPSC‐NPCs in the stroke‐damaged brain

**Figure 2:**
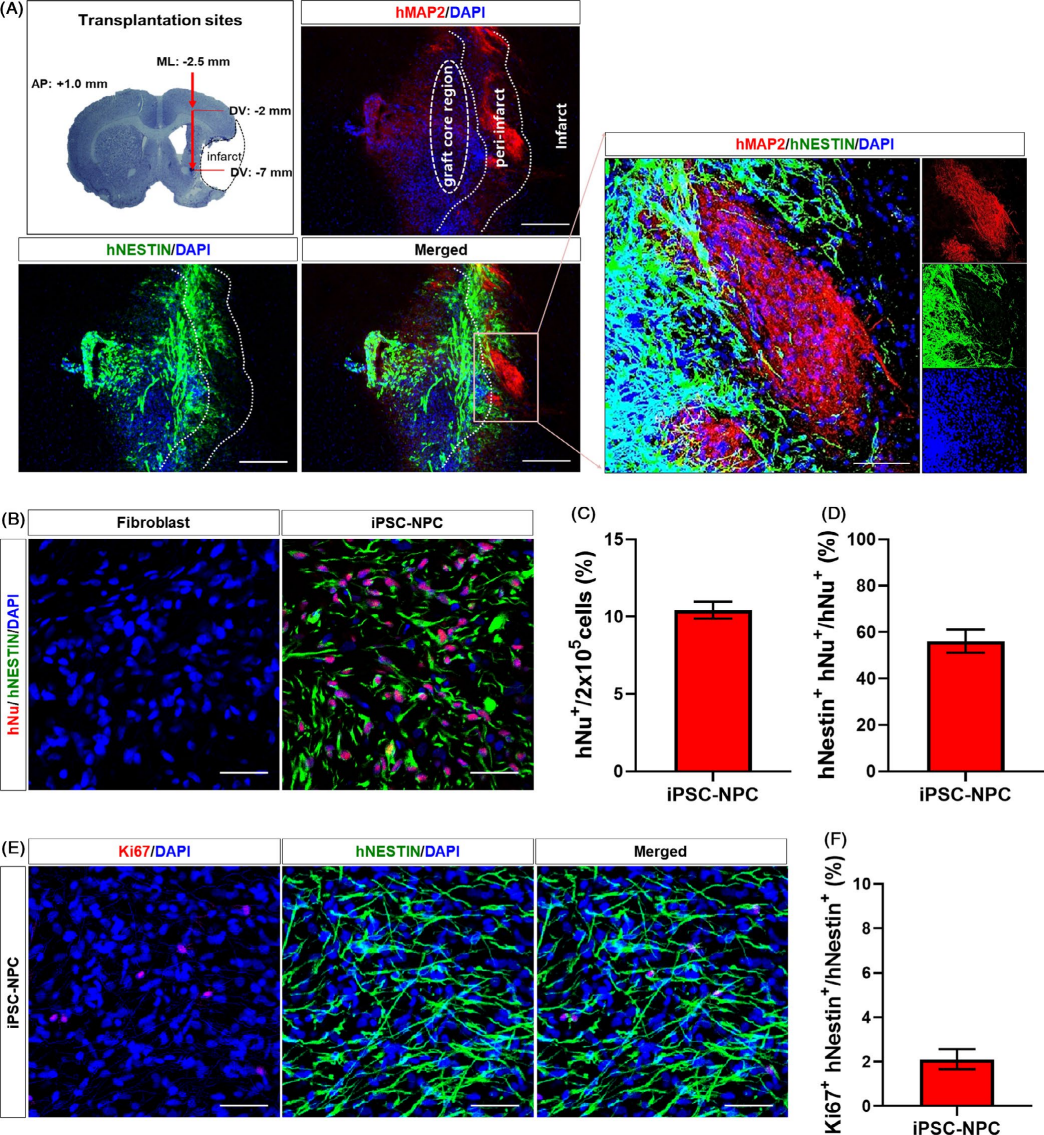
Survival and engraftment of transplanted hiPSC‐NPCs in MCAo rats. A, Representative images of double IHC for hNestin and hMAP2. The cells were transplanted into two different sites of the ipsilateral medial striatum. Note that the hNestin^+^ cells (ie undifferentiated NPCs) were mainly located in the graft site, whereas hMAP2^+^ cells (ie differentiated neurons) were located outside the graft core towards the peri‐infarct area. B, Other representative images of double IHC for hNu and hNestin from the Fibroblast and iPSC‐NPC groups. C, Quantitative analysis of hNu^+^ cells in the iPSC‐NPC group. D, Quantitative analysis of hNu^+^‐hNestin^+^ cells in the iPSC‐NPC group. Seven animals per group were used for the comparative analysis of hNu^+^ and hNu^+^‐hNestin^+^ cells. E, Representative images of double IHC for hNestin and Ki67 in the iPSC‐NPC group. F, Quantitative analysis of hNestin^+^‐Ki67^+^ cells in the iPSC‐NPC group. Five animals per group were used for the comparative analysis of hNestin^+^‐Ki67^+^ cells. DAPI was used to counterstain the nuclei. Data are expressed as the mean ± SEM. Scale bars = 50 µm

We next investigated whether transplanted hiPSC‐NPCs were able to survive and engraft after transplantation. At 12 weeks following transplantation, we observed that a significant proportion of transplanted cells were engrafted at the injection sites in the iPSC‐NPC group (Figure S2). In the core region, most of the transplanted cells were undifferentiated Nestin^+^ NPCs (Figure 2A,B). However, outside the graft core towards the infarct region, a considerable proportion of transplanted cells were differentiated into hMAP2^+^ mature neurons (Figure 2A). While no transplanted cells were detected in the Fibroblast group, hNu^+^ and hNestin^+^ cells were clearly engrafted at 12 weeks following transplantation (Figure 2B). Quantitative analysis of DAB immunostaining for hNu revealed that a total of 20 846 ± 1087 hNu^+^ cells were detected, which accounted for 10.42 ± 0.54% of transplanted cells that were engrafted at this time point (Figure 2C). Although precise quantitative analysis of the hNestin^+^ cells was not readily feasible, given the high density of cell bodies and their projecting fibres,^42^ we estimated that approximately 56.10 ± 5.01% of the transplanted cells merged with hNu and hNestin, indicating that a significant proportion of transplanted cells still remained as neural precursor cells at 12 weeks after transplantation (Figure 2D). To examine the proliferative activity of engrafted cells, we performed immunohistochemistry for Ki67, a marker of proliferating cells, and observed that 2.12 ± 0.45% of the cells were positive, indicating that most of the engrafted NPCs remained in a non‐proliferating state (Figure 2E,F).

### 3.4 Neuronal and glial differentiation of transplanted hiPSC‐NPCs in the stroke‐damaged brain

**Figure 3:**
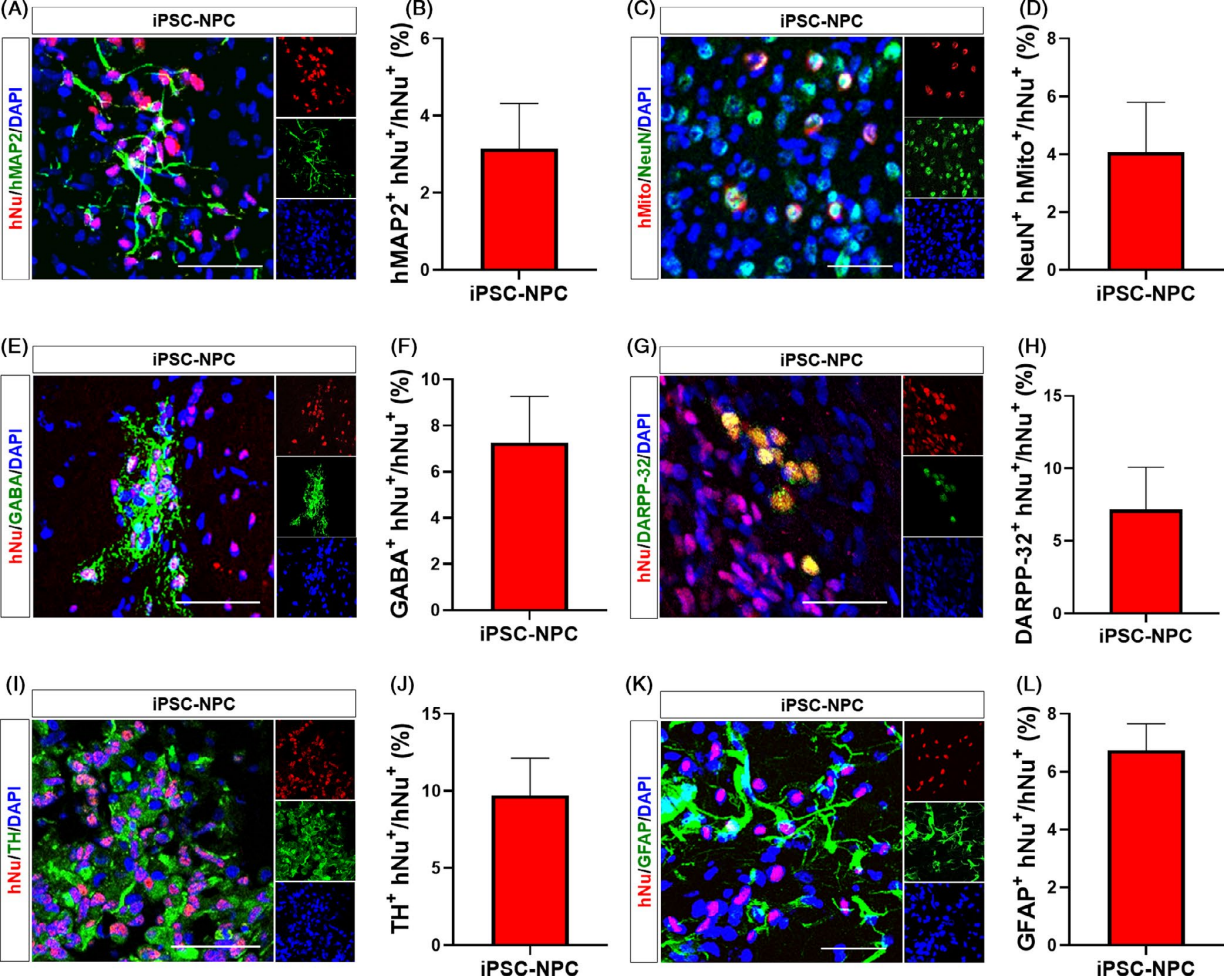
Neuronal differentiation of transplanted hiPSC‐NPCs at the peri‐infarct area in MCAo rats. A, Representative images of double IHC for hNu and hMAP2. B, Quantitative analysis of hNu^+^‐hMAP2^+^ cells in the iPSC‐NPC group. C, Representative images of double IHC for hMito and NeuN. D, Quantitative analysis of hMito^+^‐NeuN^+^ cells in the iPSC‐NPC group. E, Representative images of double IHC for hNu and GABA. F, Quantitative analysis of hNu^+^‐GABA^+^ cells in the iPSC‐NPC group. G, Representative images of double IHC for hNu and DARPP‐32. H, Quantitative analysis of hNu^+^‐DARPP‐32^+^ cells in the iPSC‐NPC group. I, Representative images of double IHC for hNu and TH. J, Quantitative analysis of hNu^+^‐TH^+^ cells in the iPSC‐NPC group. K, Representative images of double IHC for hNu and GFAP. L, Quantitative analysis of hNu^+^‐GFAP^+^ cells in the iPSC‐NPC group. Seven animals per group were used for IHC analysis. DAPI was used to counterstain the nuclei. Data are expressed as the mean ± SEM. Scale bars = 50 µm

We next performed quantitative analyses of the neuronal and glial differentiation from transplanted iPSC‐NPCs in the peri‐infarct area, in which more differentiated forms of transplanted cells were potentially able to induce neural replacement (Figure 3A).^42^ As a result, approximately 1329 ± 846 hNu^+^ cells in the iPSC‐NPC group were detected in the peri‐infarct area, where they were differentiated into hMAP2^+^ (Figure 3A,B) or NeuN^+^ mature neurons (Figure 3C,D). Further analysis revealed that transplanted cells were differentiated into GABAergic neurons (Figure 3E,F), DARPP‐32^+^ medium spiny neurons (Figure 3G,H) or TH^+^ dopaminergic neurons (Figure 3I,J). They were also differentiated into GFAP^+^ astroglial cells (Figure 3K,L).

### 3.5 Neuronal connection between transplanted hiPSC‐NPCs and host brain cells

**Figure 4:**
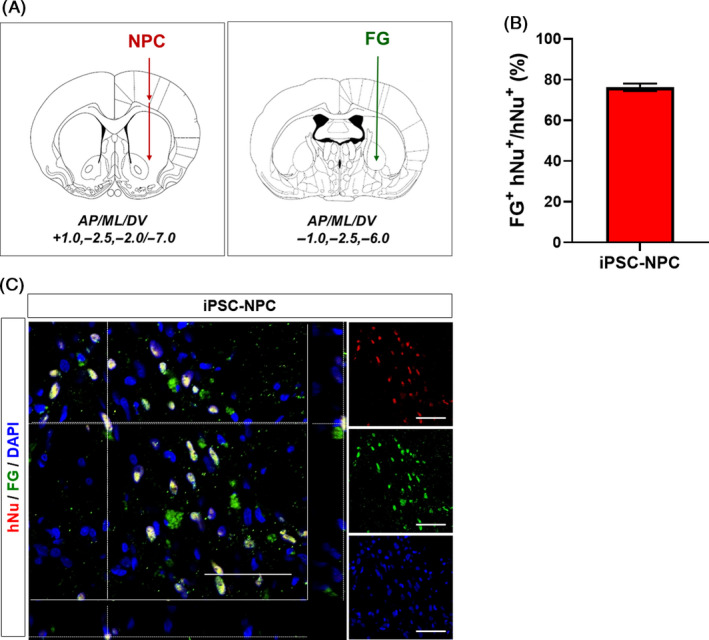
Transplanted hiPSC‐NPCs formed neural networks with host tissues in MCAo rats. A, Schematic diagrams showing the injection sites for hiPSC‐NPCs and Fluoro‐Gold (FG). The FG was injected into the area of the globus pallidus. B, Quantitative analysis of the proportion of FG^+^‐hNu^+^ cells in the striatum. Data are expressed as the mean ± SEM. C, Representative image of the co‐localization of FG^+^ (green) and hNu^+^ (red) double‐positive cells in the striatum (yellow). Three animals per group were used for neural network analysis. DAPI was used to counterstain the nuclei. Scale bars = 50 µm

To investigate whether transplanted cells can form a neuronal connection with host brain cells, we injected a retrograde neuronal tracer, Fluoro‐Gold (FG), into the globus pallidus on the ipsilateral side and analysed the co‐labelled hNu^+^‐FG^+^ cells in the ipsilateral striatum (Figure 4A). A considerably high proportion of transplanted hiPSC‐NPCs (76.22 ± 1.80%) showed positivity for FG signals (Figure 4B,C), suggesting that the transplanted human cells were successfully connected with host striatal neurons, forming a neuronal network between the transplanted cells and the host brain.

### 3.6 Reduction of host immune responses and gliosis following transplantation of hiPSC‐NPCs

**Figure 5:**
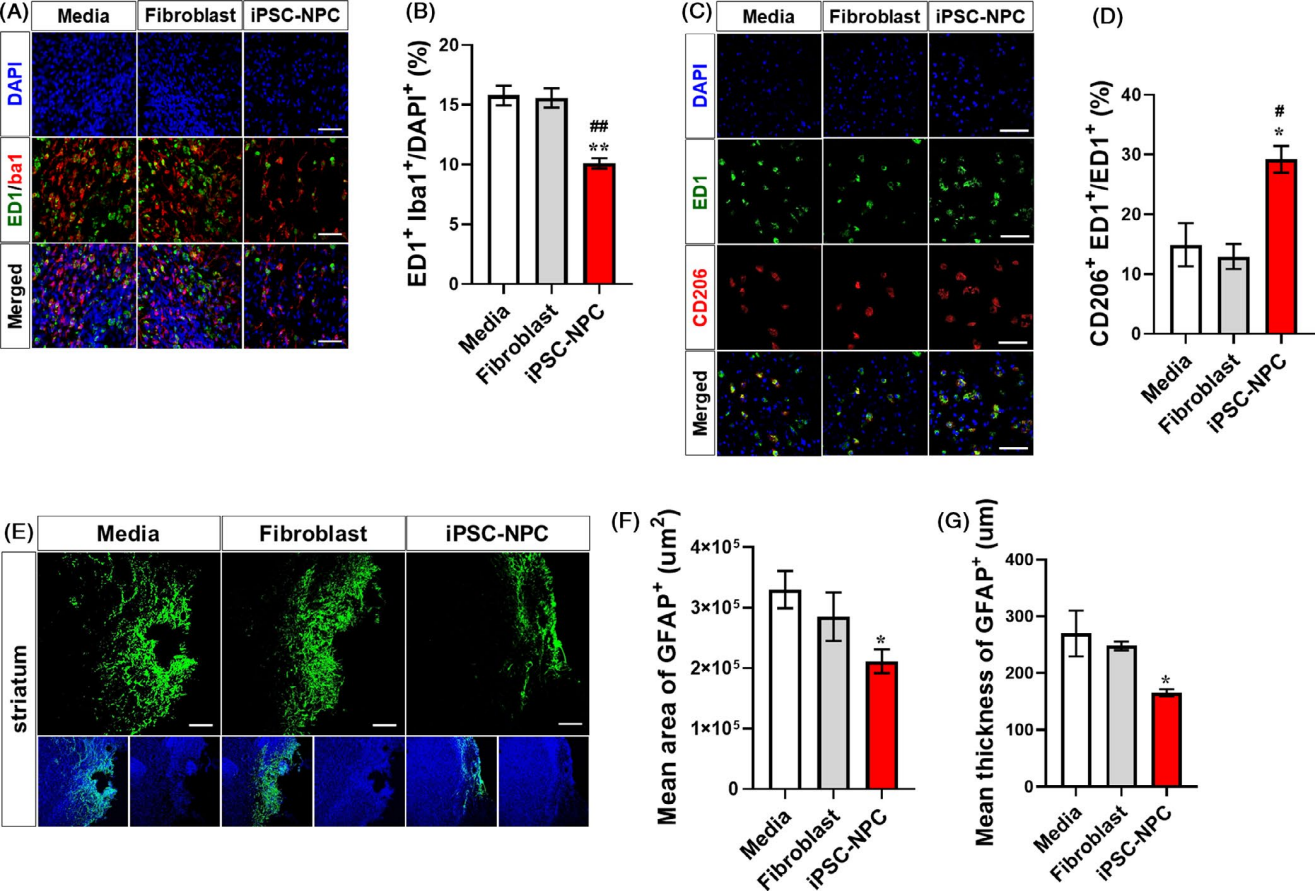
Transplanted hiPSC‐NPCs reduce aberrant post‐stroke neuroinflammation and glial scar formation in MCAo rats. A, Representative images of double IHC for ED1 and Iba1. B, Quantitative analysis of ED1^+^‐Iba1^+^ cells in the Medium, Fibroblast and iPSC‐NPC groups. C, Representative images of double IHC for ED1 and CD206. D, Quantitative analysis of ED1^+^‐CD206^+^ cells in the Medium, Fibroblast and iPSC‐NPC groups. E, Representative images of IHC for GFAP (green) at the peri‐infarct area. F, Quantitative analysis of mean area of GFAP^+^ glial scar at the peri‐infarct area in the Medium, Fibroblast and iPSC‐NPC groups. G, Quantitative analysis of mean thickness of GFAP^+^ glial scar at the peri‐infarct area in the Medium, Fibroblast and iPSC‐NPC groups. Five animals per group were used for IHC analysis. DAPI was used to counterstain the nuclei. Data are expressed as the mean ± SEM Statistical significance by one‐way ANOVA with Tukey's *b* method is shown as follows: for Medium vs iPSC‐NPC: *P < .05; **P < .01; for Fibroblast vs iPSC‐NPC, ^#^ *P *< .05; ^##^ *P *< .01. Scale bars = 50 µm

To understand the changes of neuroinflammation following transplantation, we investigated the extent of microglial activation and glial scar formation. In the brain of MCAo rats, numerous microglial cells (Iba1^+^ cells) were found in the peri‐infarct area, some of which were in activated form (ED1^+^ cells). We observed that the activated phagocytic ED1^+^‐Iba1^+^ cells were significantly reduced in the iPSC‐NPC group compared with the levels in the Medium and Fibroblast groups (Figure 5A,B). We further investigated the proportion of different microglial phenotypes by performing double immunostaining for iNOS^+^‐ED1^+^ cells and CD206^+^‐ED1^+^ cells. The iNOS‐expressing microglia/macrophages secrete pro‐inflammatory cytokines such as interleukin‐1 beta (IL‐1β) and tumour necrosis factor alpha (TNF‐α), which subsequently exacerbate brain injury in stroke.^43^ On the other hand, CD206 (also known as a mannose receptor)–expressing microglia/macrophages suppress aberrant inflammation and participate in the healing process by phagocytizing waste and dead cells in the damaged area after stroke.^44^ As a result, the proportion of CD206^+^‐ED1^+^ cells was significantly increased in the iPSC‐NPC group compared with the levels in the Medium and Fibroblast groups (Figure 5C,D). The proportion of iNOS^+^‐ED1^+^ cells was not significantly different among the three groups (Figure S3A,B). These findings suggest that the transplantation of hiPSC‐NPCs not only ameliorates post‐stroke neuroinflammation but also promotes the healing process in the damaged brain.

Next, we evaluated the glial scar area and the thickness of the ipsilateral hemisphere by performing immunostaining using an antibody against GFAP (Figure 5E). In the peri‐infarct cortex, no difference was observed in the area and thickness of GFAP^+^ glial scar among the three groups (Figure S4A‐C). However, in the peri‐infarct striatal area, the area of glial scar was reduced in the iPSC‐NPC group compared with that in the Medium and Fibroblast groups (Figure 5F). The thickness of the glial scar was also reduced in the iPSC‐NPC group compared with the levels in the Medium and Fibroblast groups (Figure 5G). These findings suggest that the transplantation of hiPSC‐NPCs not only promotes the healing process after post‐stroke neuroinflammation but also prevents glial scar formation in the subacute phase of ischaemic stroke in rats.

### 3.7 Increased endogenous neurogenesis following transplantation of hiPSC‐NPCs

**Figure 6:**
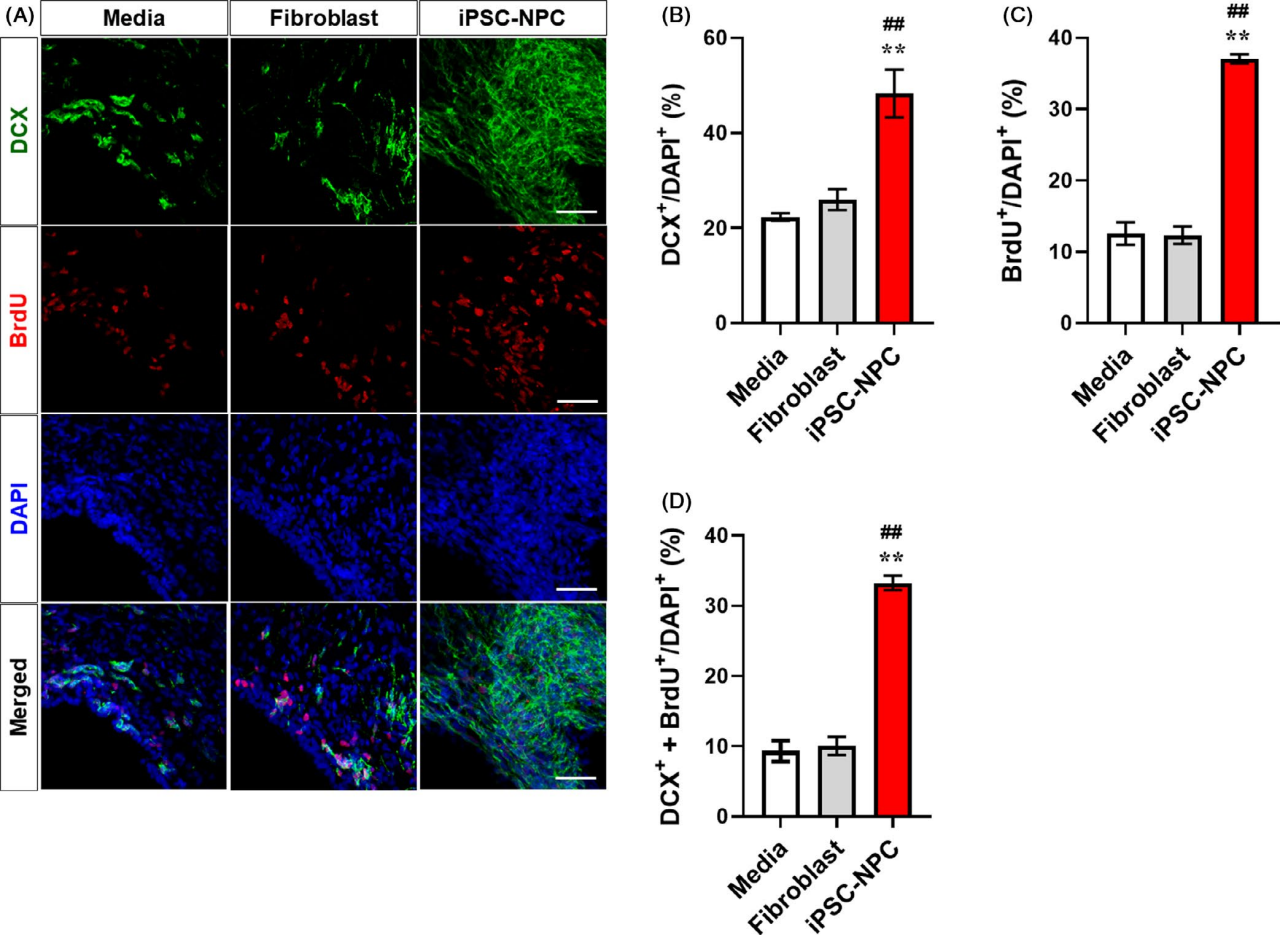
Transplanted hiPSC‐NPCs increase endogenous neurogenesis in MCAo rats. A, Representative images of double IHC for DCX and BrdU. B, Quantitative analysis of DCX^+^ cells in the Medium, Fibroblast and iPSC‐NPC groups. C, Quantitative analysis of BrdU^+^ cells in the Medium, Fibroblast and iPSC‐NPC groups. D, Quantitative analysis of DCX^+^‐BrdU^+^ cells in the Medium, Fibroblast and iPSC‐NPC groups. Five animals per group were used for IHC analysis. DAPI was used to counterstain the nuclei. Data are expressed as the mean ± SEM. Statistical significance by one‐way ANOVA with Tukey's *b* method is shown as follows: for Medium vs iPSC‐NPC: *P < .05; **P < .01; for Fibroblast vs iPSC‐NPC: ^#^ *P *< .05; ^##^ *P *< .01

In ischaemic brain injury, endogenous neural progenitor cells generated in the subventricular zone (SVZ) are known to migrate towards the injury site to replace the lost brain cells. To investigate whether transplanted cells can affect the endogenous SVZ neurogenesis, we performed double staining for BrdU and DCX to detect proliferating neural progenitor cells (Figure 6A). Immunohistochemical staining revealed that the number of DCX^+^ neuroblasts was significantly increased in the iPSC‐NPC group compared with the levels in the Medium and Fibroblast groups (Figure 6B). In addition, the number of BrdU^+^ proliferating cells in the ipsilateral SVZ was significantly increased in the iPSC‐NPC group compared with those in the Medium and Fibroblast groups (Figure 6C). In particular, the number of BrdU and DCX double‐positive cells (ie proliferating neuroblasts) was significantly increased in the iPSC‐NPC group compared with the levels in the Medium and Fibroblast groups (Figure 6D). These results strongly suggest that the transplantation of hiPSC‐NPCs enhances SVZ neurogenesis in the damaged brain following stroke.

### 3.8 Increased perilesional angiogenesis following transplantation of hiPSC‐NPCs

**Figure 7:**
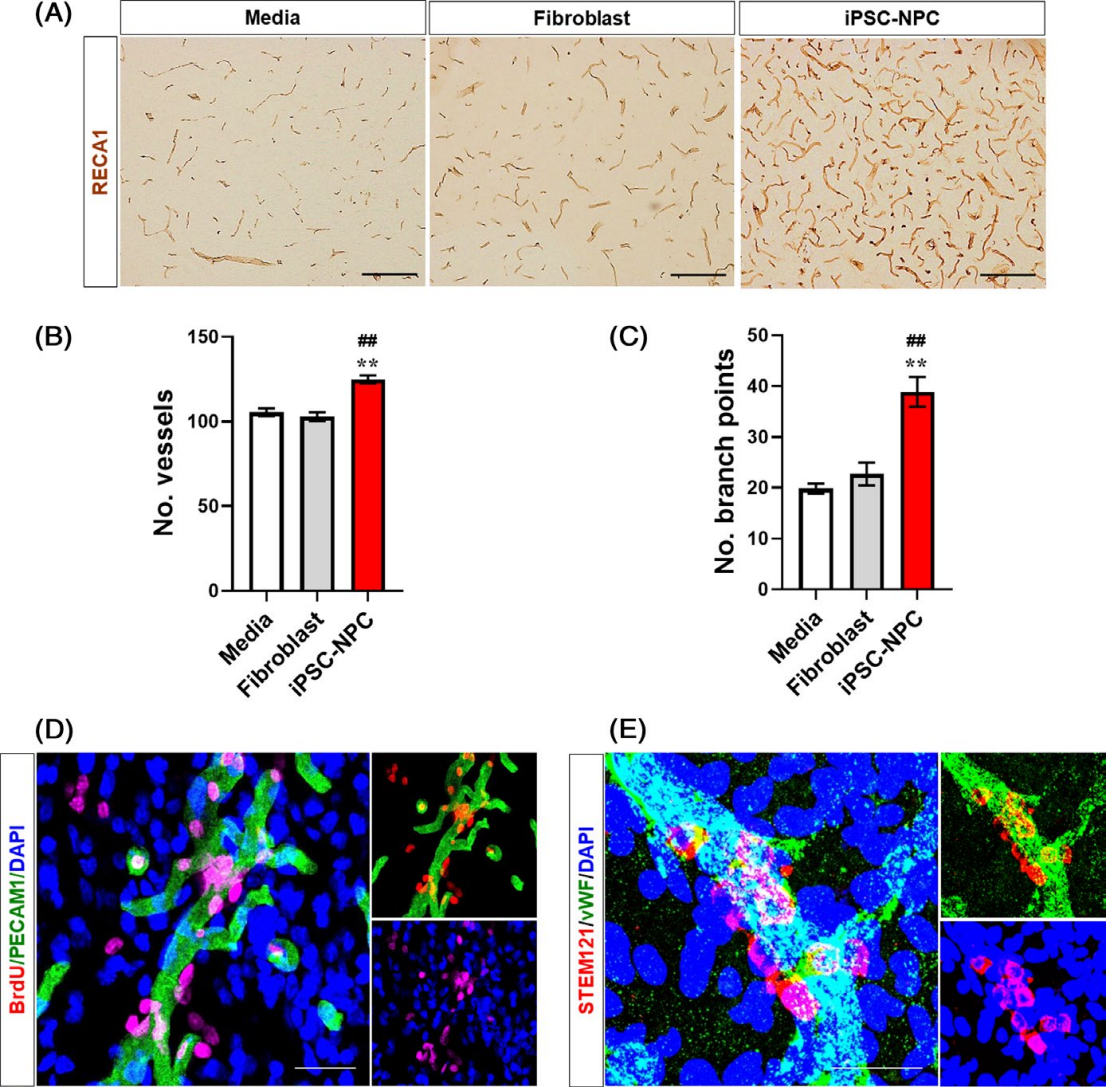
Transplanted hiPSC‐NPCs increase peri‐infarct angiogenesis in MCAo rats. A, Representative images of DAB immunostaining for RECA‐1. B, Quantitative analysis of the number of peri‐infarct blood vessels in the Medium, Fibroblast and iPSC‐NPC groups. C, Quantitative analysis of the number of branch points of peri‐infarct blood vessels in the Medium, Fibroblast and iPSC‐NPC groups. Five animals per group were used for IHC analysis. Data are expressed as the mean ± SEM. Statistical significance by one‐way ANOVA with Tukey's b method is shown as follows: for Medium vs iPSC‐NPC: *P < .05; **P < .01; for Fibroblast vs iPSC‐NPC: ^#^ *P *< .05; ^##^ *P *< .01. D, Representative images of double IHC for BrdU and PECAM1. Note that the proliferating cells (ie BrdU^+^ cells) were predominantly located around the vessel walls (ie PECA1^+^ vessels) at the peri‐infarct area. E, Representative images of double IHC for STEM121 and vWF. Note that a considerable number of the transplanted hiPSC‐NPCs (ie STEM121^+^ cells) were located around the RECA1^+^ endothelial cells at the peri‐infarct area. DAPI was used to counterstain the nuclei. Scale bars = 100 µm (A), 50 µm (D and E)

We also investigated whether the transplantation of hiPSC‐NPCs can induce new vessel formation in the perilesional area following stroke (Figure 7A). Immunostaining results indicated that the number of RECA1^+^ blood vessels (<30 µm in diameter) in the peri‐infarct area was significantly increased in the iPSC‐NPC group compared with the levels in the Medium and Fibroblast groups (Figure 7B). The number of branch points in the capillaries was also significantly increased in the iPSC‐NPC group compared with those in the Medium and Fibroblast groups (Figure 7C). In addition, we observed that BrdU^+^ proliferating cells were present around the PECA1^+^ vessels (Figure 7D). Interestingly, we observed that transplanted hiPSC‐NPCs, shown as STEM121^+^ cells, were present around RECA1^+^ endothelial cells in the peri‐infarct area (Figure 7E). These results strongly suggest that transplanted hiPSC‐NPCs are actively involved in the repair of peri‐infarct blood vessels and promote angiogenesis following stroke in rats.

## 4 DISCUSSION

This study demonstrates that the intracerebral transplantation of HLA‐homozygous human iPSC‐derived neural precursors (hiPSC‐NPCs) can ameliorate the behavioural and pathological deficits in a rodent model of ischaemic stroke. The advantages of using HLA‐homozygous hiPSCs for cell therapy lie in their capacity to be transplanted into large numbers of HLA‐matched patients without or with minimal risk of immune rejection.^22^, ^45^ We recently generated the top ten common HLA‐homozygous iPSCs that, in total, cover 41.7% of the South Korean population.^23^ Similar results have been reported for the UK^45^ and Japanese populations,^46^ highlighting the significance of HLA‐homozygous iPSC‐based approaches. Although 'universal cells' that may escape allogeneic responses have recently been introduced by knocking out and manipulating several HLA‐related genes,^47^, ^48^ there are still many safety‐related issues to be resolved before they are entered into human trials.^49^

In this study, we investigated the therapeutic effects of Good Manufacturing Practice (GMP)‐grade HLA‐homozygous hiPSCs^25^ in treating a preclinical rodent model of ischaemic stroke. The intracerebral transplantation of hiPSC‐NPCs gave rise to improved behavioural recovery in clinically relevant motor and sensory deficits. Our results support previous studies demonstrating that hiPSC‐NPC transplantation restored sensorimotor and behavioural functions in stroke‐damaged animals.^6^, ^50^, ^51^, ^52^, ^53^, ^54^ Several studies previously reported functional recovery in animals transplanted with hiPSC‐NPCs in the subacute phase of stroke (ie 1 week after stroke induction).^50^, ^51^, ^53^ This time period of transplantation is particularly important from a clinical perspective because there is currently no effective treatment at this stage or after it. Therefore, given our results and other previously reported studies, hiPSC‐NPC transplantation can be a promising therapeutic approach for patients with ischaemic stroke at the subacute phase.

The main advantage of using NPCs is their potential to replace the lost neuronal circuitry and thus to have prolonged beneficial effects. Histological analyses performed at 12 weeks post‐transplantation in this study indicated that transplanted hiPSC‐NCPs survived and differentiated into various neuronal phenotypes, including hMAP2^+^, NeuN^+^, GABA^+^, DARPP‐32^+^ and TH^+^ neurons in vivo. The differentiation rate of transplanted NPCs was highly variable in the previous studies, showing that 5%‐70% of transplanted NPCs derived from iPSCs or hESCs differentiated into various types of mature neuron. By contrast, only a small percentage (ie <6%) were differentiated into glial cells,^6^, ^7^, ^42^, ^50^, ^51^, ^55^, ^56^, ^57^, ^58^, ^59^ in agreement with our results. To confirm the functionality of differentiated neurons, it will be extremely important to perform electrophysiological studies in future work. Nevertheless, our retrograde neuronal tracing analysis showed the capacity of transplanted hiPSC‐derived neurons to connect to striatal neurons in the host brain, demonstrating that differentiated hiPSC‐derived neurons can potentially integrate with the host neural network.

The risk of tumour formation is a major concern for pluripotent stem cell‐based transplantation therapies, especially when the transplanted NPCs remain in an undifferentiated state. However, previous studies demonstrated that, although significant proportions of engrafted NPCs remained as Nestin^+^ undifferentiated neural precursor cells, they rarely expressed Ki‐67 (ie 1%‐2% of transplanted cells), indicating that the great majority of transplanted NPCs were non‐proliferative.^42^, ^50^, ^53^, ^55^ The exact mechanism behind this phenomenon is unclear, but it is possible that the transplanted NPCs have the propensity to retain their original characteristics. Another possible explanation is that microenvironment in the stroke‐damaged brain may render the transplanted NPCs unable to proliferate but able to differentiate into appropriate neurons. During the 12 weeks after transplantation, we did not detect any morphological signs of tumour formation in vivo. In agreement with previous findings, the proportion of Ki67^+^ cells was extremely low, indicating the low risk of proliferation of transplanted cells. However, a potential risk of tumorigenicity beyond the duration of our experiment cannot be fully ruled out. Therefore, in future work, long‐term follow‐up study of up to 6 or 12 months will be necessary to clarify the possibility of tumorigenicity of transplanted NPCs.

On the other hand, it was recently shown that non‐proliferating, undifferentiated engrafted NPCs play a role in functional recovery due to their bystander effects by releasing immunomodulatory and neurotrophic factors.^10^, ^19^, ^60^, ^61^, ^62^ Therefore, we investigated whether transplanted cells can attenuate the stroke‐induced inflammatory/immune response.^63^ We observed a significant reduction of activated ED1‐positive microglia and the increase of the healing process of post‐stroke neuroinflammation at 12 weeks after transplantation. Furthermore, we observed that the area and thickness of glial scar were significantly reduced by transplanted hiPSC‐NPCs. These findings are attributable to the bystander effects of grafted cells by releasing immunomodulatory factors, in order to promote the healing process and to prevent glial scar formation. The immunomodulatory actions of NPCs have also been observed when they were systemically delivered in stroke animal models,^10^, ^64^ supporting our findings.

In the adult brain, cerebral ischaemia induces SVZ neurogenesis and the newly generated neuroblasts migrate to the sites of injury.^65^, ^66^, ^67^ However, this self‐repair process is highly limited in terms of enabling the damaged brain to achieve significant functional recovery following stroke.^68^ To assist this process, the transplantation of NPCs is expected to enhance endogenous repair.^64^, ^69^, ^70^ Supporting this expectation, we demonstrated that the transplantation of hiPSC‐NPCs significantly increased the number of proliferating cells (BrdU^+^) and endogenous NPCs (DCX^+^) in the ipsilateral SVZ. In particular, the increase of BrdU^+^‐DCX^+^ proliferating neuroblasts strongly suggested that the transplantation of hiPSC‐NPCs enhanced the endogenous neurogenesis and the migration of newly generated neuroblasts towards the ischaemic lesion. Interestingly, neurogenesis is coupled with angiogenesis in ischaemic stroke.^71^, ^72^ Stroke‐induced angiogenesis in the ischaemic penumbra region is known to provide scaffolds to guide neuroblasts to the lesion.^72^ It is known that the cerebral endothelial cells are relatively quiescent in the SVZ niche.^73^, ^74^ Our study showed that the transplantation of hiPSC‐NPCs increased the number and branches of peri‐infarct blood vessels, which is consistent with previous studies.^62^, ^75^ We observed a significant increase of PECAM^+^/BrdU^+^ proliferating vessels in the ischaemic area after the transplantation of hiPSC‐NPCs. Interestingly, we observed that some NPCs were located close to the cerebral endothelial cells, indicating the direct incorporation of transplanted cells into the newly formed blood vessels.

In summary, we demonstrated that the transplantation of HLA‐homozygous hiPSC‐NPCs can give rise to therapeutic effects in an animal model of subacute stroke. The transplanted cells were successfully differentiated into various neuronal cell types and connected to the host neurons. In addition, the transplanted cells promoted the endogenous process of brain repair, such as the increases of SVZ neurogenesis and angiogenesis, and the decreases of neuroinflammation and glial scar formation. Given that HLA‐homozygous hiPSCs have many important advantages over autologous hiPSCs in clinical practice, our study provides strong preclinical evidence for the clinical application of HLA‐matched hiPSC therapy in patients with ischaemic stroke in the near future.

## CONFLICT OF INTEREST

JS is the founder and CEO of iPS Bio, Inc The other authors declare no conflicts of interests.

## AUTHOR CONTRIBUTIONS

JS, J.‐EN and S.‐HO conceived the study and designed the experiments. J.‐EN, SL, SL and YHK performed the experiment. JHJ provided CMC‐hiPSC‐004 for transplantation experiment. J.‐EN, S.‐HO, HJP, HSK and JYH analysed the results and contributed to the writing of manuscript. JS provided funding for the work and wrote the manuscript. JS supervised the entire work and approved the final submission of manuscript. J.‐EN and S.‐HO contributed equally.

## Supporting information

Supplementary Material

## Data Availability

The data that support the findings of this study are available from the corresponding author upon reasonable request.
