# Supplementary material for: Intracerebral transplantation of HLA‐homozygous human iPSC‐derived neural precursors ameliorates the behavioural and pathological deficits in a rodent model of ischaemic stroke
Source: Cell Prolif. 2020 Jul 26;53(9):e12884. doi: 10.1111/cpr.12884 (PMC7507302; doi:10.1111/cpr.12884)
Supplement: Supplementary file 1 — Supplementary Material [file CPR-53-e12884-s001.docx]

**Supporting information file**

**Cell Proliferation**

**Intracerebral transplantation of HLA-homozygous human iPSC-derived neural precursors ameliorates the behavioral and pathological deficits in a rodent model of ischemic stroke**

Jeong-Eun Noh, Seung-Hun Oh, Suji Lee, Soohyeon Lee, Young Hoon Kim, Hyun Jung Park, Ji Hyeon Ju, Hyun Sook Kim, Ji Young Huh, and Jihwan Song

**Contents**

Figure S1. Characterization of hiPSC-NPC and their neuronal differentiation *in vitro*

Figure S2. Survival and engraftment of transplanted hiPSC-NPCs in MCAo rats

Figure S3. Changes of post-stroke neuroinflammation by transplanted hiPSC-NPCs

Figure S4. Changes of glial scar formation in the peri-infarct cortex by transplanted hiPSC-NPCs in MCAo rats

Table S1. List of primary antibodies used for immunocytochemical staining

Table S2. List of primary antibodies used for immunohistochemical staining

**Supplementary Figures and Legends**

**
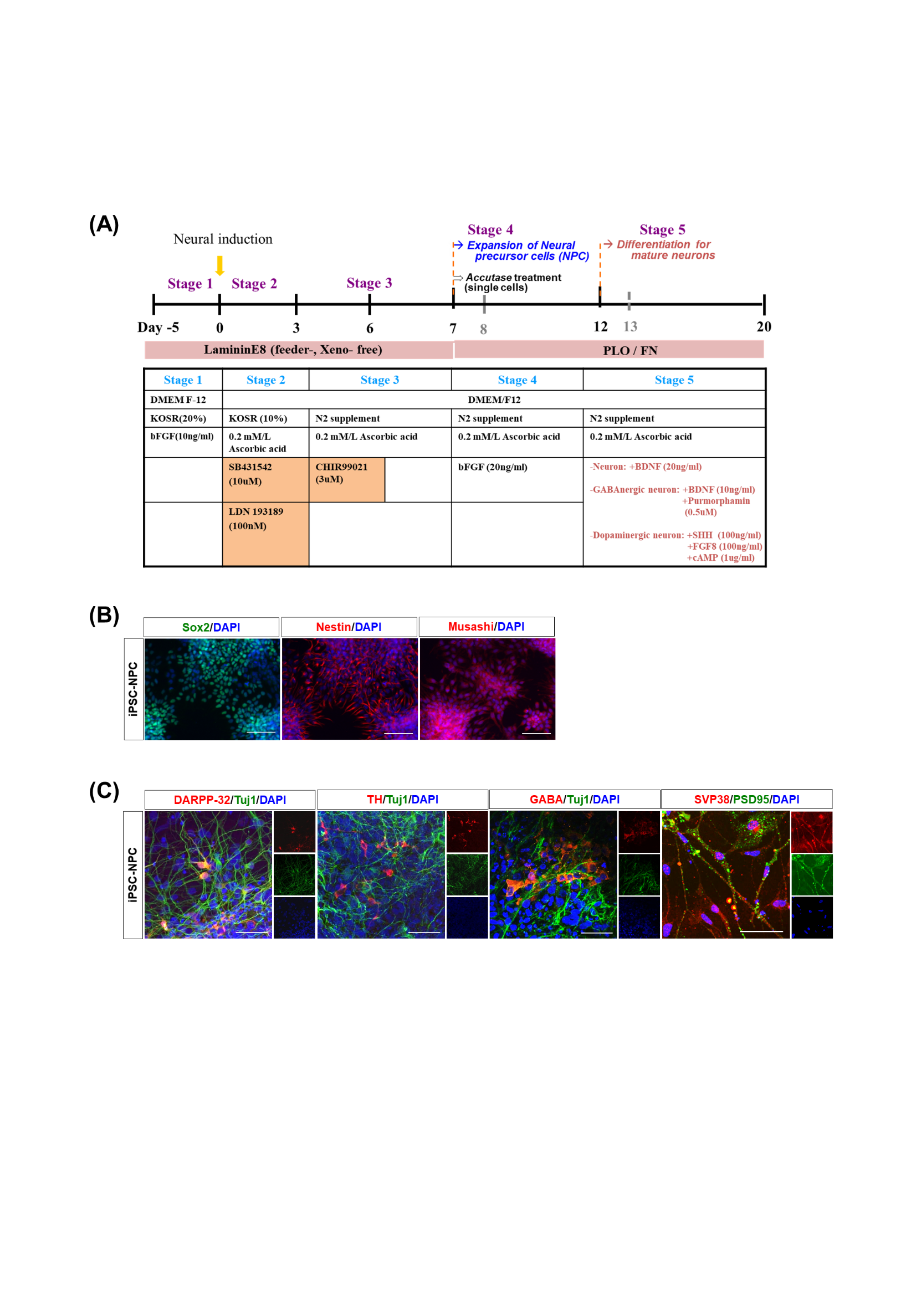
**

**Figure S1. Characterization of hiPSC-NPC and their neuronal differentiation in vitro**

**(A)** Schematic diagram showing the differentiation procedures of hiPSCs into NPCs and mature neurons. **(B)** Representative immunocytochemical images hiPSCs-NPCs. The NPCs expressed Musashi, Nestin, and Sox2. Scale bar = 100 μm. **(C)** Representative immunocytochemical images of hiPSCs-NPCs-derived mature neurons. The hiPSC-NPCs-derived Tuj1^+^ neurons expressed DARRP-32, TH and GABA. Furthermore, the hiPSC-NPCs-derived neurons expressed synaptic vesicle markers, such as SVP38 and PSD95. indicating synaptic formation of differentiated neurons. Scale bar = 50 μm.

**
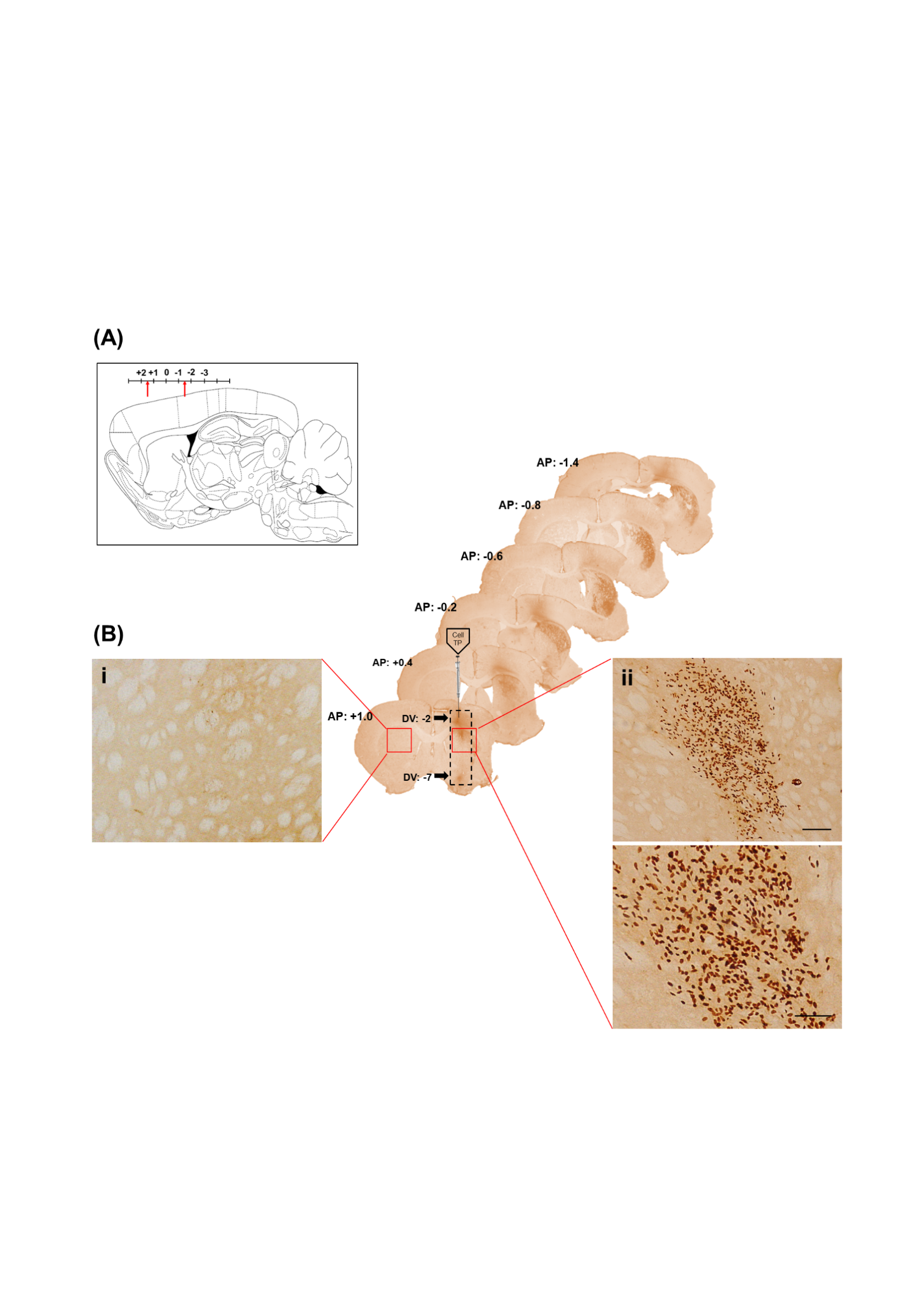
**

**Figure S2. Survival and engraftment of transplanted hiPSC-NPCs in MCAo rats**

**(A)** A schematic diagram of tissue analysis in rat brain. Red arrows indicate the range of brain areas for analysis (between +1.5 and -1.5 from bregma). **(B)** Representative image of hNu DAB immunostaining of transplanted hiPSC-NPCs in MCAo rats at 12 weeks after transplantation. While there was no cell detected in contralateral striatum (i), hNu+ cells (dark brown) were detected in the ipsilateral side of infarction (ii and iii). Scale bar = 100 μm. AP: anteroposterior, DV: dorsoventral.


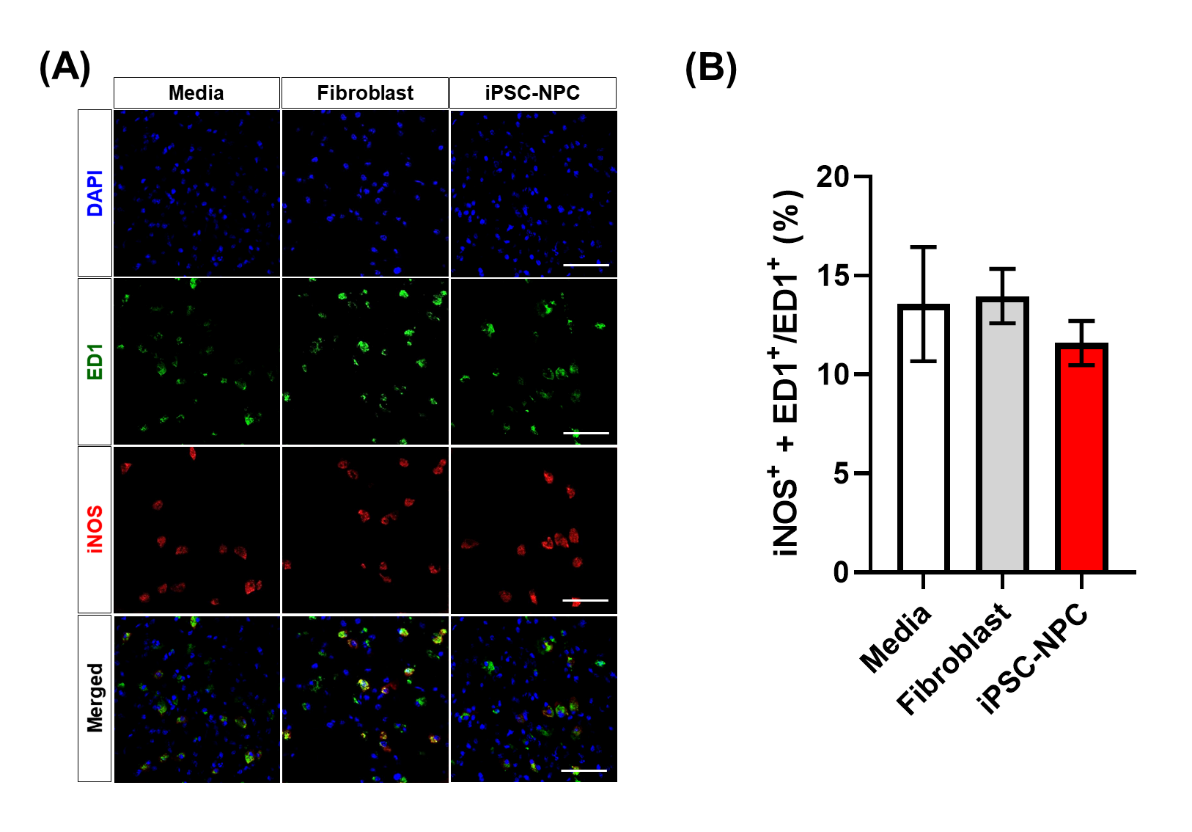


**Figure S3. Changes of post-stroke neuroinflammation by transplanted hiPSC-NPCs**

**(A)** Representative images of double IHC for ED1 and iNOS. DAPI was used to counterstain the nuclei. Scale bar = 50 μm. **(B)** Quantitative analysis of the proportion of iNOS^+^-ED1^+^ cells in the Media, Fibroblast, and iPSC-NPC groups. Data are expressed as the mean ± SEM. Statistical analysis was performed using the one-way ANOVA with Tukey’s b method.


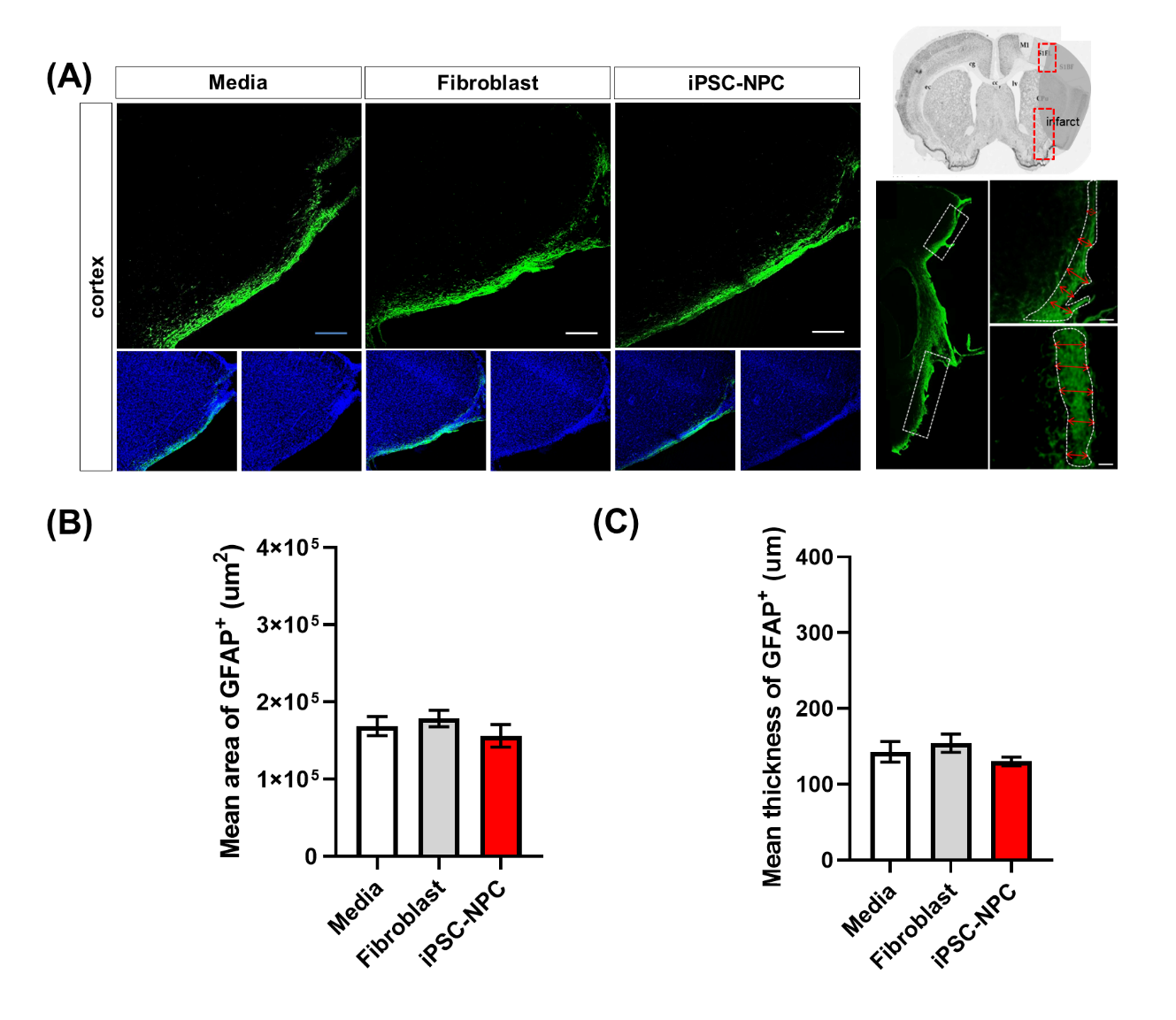


**Figure S4. Changes of glial scar formation in the peri-infarct cortex by transplanted hiPSC-NPCs in MCAo rats**

**(A)** Representative image of GFAP immunostaining in the peri-infarct cortex in MCAo rats. Right upper panel represents the area of analysis. Right lower panel represents the mean thickness of GFAP glial scar, which is defined as average of thickness on five areas. The white-dotted areas indicate the area of glial scar. DAPI was used to counterstain the nuclei. Scale bar = 100 μm. **(B)** Quantitative analyses of the area of GFAP^+^ glial scar in the Media, Fibroblast and iPSC-NPC groups. **(C)** Quantitative analyses of the mean thickness of GFAP^+^ glial scar in the Media, Fibroblast and iPSC-NPC groups. Data are expressed as the mean ± SEM. Statistical analysis was performed using the one-way ANOVA with Tukey’s b method.

**Table S1. List of primary antibodies used for immunocytochemical staining**

| Primary antibodies | Dilution | Host species | Suppliers | Catalogue number |
| --- | --- | --- | --- | --- |
| Sox2 | 1:200 | rabbit | Millipore | AB5603 |
| Nestin | 1:200 | rabbit | R&D System | 841901 |
| Musashi | 1:200 | rabbit | Millipore | AB5977 |
| Tuj1 | 1:500 | mouse | Millipore | MAB1637 |
| GABA | 1:500 | rabbit | Sigma-Aldrich | A2052 |
| DARPP-32 | 1:200 | rabbit | Cell Signaling | MBS9407621 |
| TH | 1:200 | rabbit | Pel-Freez | P40101-150 |
| SVP38 | 1:200 | mouse | Abcam | ab8049 |
| PSD95 | 1:200 | rabbit | Cell Signaling | 2507s |

**Table S2. List of primary antibodies used for immunohistochemical staining**

| Primary antibodies | Dilution | Host species | Suppliers | Catalogue number |
| --- | --- | --- | --- | --- |
| Human nuclei (hNu) | 1:100 | mouse | Millipore | MAB1281 |
| Human mitochondria (hmito) | 1:100 | rabbit | Millipore | AB3598 |
| STEM121 | 1:250 | mouse | Takara | Y40410 |
| hNestin | 1:200 | rabbit | R&D System | 841901 |
| hNestin | 1:250 | mouse | Abcam | ab22035 |
| Ki67 | 1:200 | mouse | Invitrogen | 14-5699-82 |
| GABA | 1:250 | rabbit | Sigma-Aldrich | A2052 |
| DARPP-32 | 1:250 | rabbit | Cell Signaling | MAB9407621 |
| TH | 1:250 | rabbit | Pel-Freez | P40101-150 |
| hMap2 | 1:250 | rabbit | Santa Cruz | SC-20172 |
| NeuN | 1:250 | mouse | Millipore | MAB377 |
| GFAP | 1:500 | mouse | BD Biosciences | MAB2594 |
| GFAP | 1:250 | rabbit | Dako | Z033429 |
| Iba1 | 1:250 | rabbit | Wako | 019-19741 |
| ED1 | 1:250 | mouse | Serotec | MCA341R |
| CD206 | 1:200 | goat | Santa Cruz | SC-34577 |
| iNOS | 1:200 | rabbit | Santa Cruz | SC-649 |
| BrdU | 1:200 | mouse | BD Biosciences | 555627 |
| DCX | 1:250 | rabbit | Cell Signaling | 4604S |
| RECA1 | 1:250 | mouse | Abcam | Ab9774 |
| PECAM1 | 1:200 | goat | R&D Systems | AF3628 |
| vWF | 1:200 | rabbit | Invitrogen | PA5-104687 |
